# Supplementary material for: Electron paramagnetic resonance as a tool to determine the sodium charge storage mechanism of hard carbon
Source: Nat Commun. 2024 Apr 8;15:3013. doi: 10.1038/s41467-024-45460-3 (PMC11001870; doi:10.1038/s41467-024-45460-3)
Supplement: Supplementary file 1 — Supplementary Information [file 41467_2024_45460_MOESM1_ESM.docx]

Supporting Information

**Electron Paramagnetic Resonance as a Tool to Determine the Sodium Charge Storage Mechanism of Hard Carbon**

Bin Wang^1,2^, Jack R. Fitzpatrick^1,2,3^, Adam Brookfield^4^, Alistair J. Fielding^5^, Emily Reynolds^6^, Jake Entwistle^1^, Jincheng Tong^7^, Ben F. Spencer^8^, Sara Baldock^1^, Katherine Hunter^9^, Christopher M. Kavanagh^9^, Nuria Tapia-Ruiz^1,2,3^*

1. Department of Chemistry, Lancaster University, Lancaster LA1 4YB, UK

2. The Faraday Institution, Harwell Science and Innovation Campus, Quad One, Didcot, OX11 0RA, UK

3. Department of Chemistry, Molecular Sciences Research Hub, White City Campus, Imperial College London, London W12 0BZ, UK

4. The National Research Facility for Electron Paramagnetic Resonance, Photon Science Institute, University of Manchester, Oxford Road, Manchester M13 9PL, UK

5. Centre for Natural Products Discovery, School of Pharmacy and Biomolecular Sciences, Liverpool John Moore University, Byrom Street, Liverpool, L3 3AF, UK

6. ISIS Neutron and Muon Spallation Source, STFC Rutherford Appleton Laboratory, Harwell, Oxford OX11 0QX, UK

7. Department of Chemistry, University of Manchester, Oxford Road, Manchester M13 9PL, UK

8. Department of Materials, University of Manchester, Oxford Road, Manchester M13 9PL, UK

9. Deregallera Ltd, Unit 2 De Clare Court, Pontygwindy Industrial Estate, Caerphilly, Wales, CF83 3HU, UK

*Corresponding Author: Dr Nuria Tapia-Ruiz. Email: n.tapia-ruiz@imperial.ac.uk

**Table of Contents**

List of Supplementary Tables……………………………………………………………….page 2

List of Supplementary Figures………………………………………………………………page 6

Supplementary References………………………………………………………………….page 15

**List of Supplementary Tables**

**Table S1. Positions of the (002) reflection (in 2θ degrees) and FWHM values obtained from XRD data of pristine and ball-milled HC samples.** Fittings were performed on the XRD data shown in Figure S3. The errors shown are the standard error output from the fitting. An example of such a fitting can be seen in Figure S4.

| Sample | (002)/° 2θ | FWHM/2θ |
| --- | --- | --- |
| HC700 | 23.7 ± 0.18 | 7.5 ± 0.4 |
| HC700-400-2h | 23.9 ± 0.11 | 7.6 ± 0.3 |
| HC700-400-5h | 24.06 ± 0.011 | 7.93 ± 0.03 |
| HC1000 | 24.2 ± 0.13 | 6.9 ± 0.3 |
| HC1000-400-2h | 24.1 ± 0.17 | 7.2 ± 0.4 |
| HC1000-400-5h | 23.872 ± 0.004 | 7.98 ± 0.011 |

**Table S2.** **XPS fitting parameters.** Fitting parameters of the C 1*s*, O 1*s* and N 1*s* high-resolution XPS data (shown in Figures S5 and S6).

| Sample | Component | Position*  /eV | FWHM**/eV | Area | Sample | Component | Position*  /eV | FWHM**/eV | Area |
| --- | --- | --- | --- | --- | --- | --- | --- | --- | --- |
| C 1*s* | | | | | | | | | |
| HC1000 | *sp*^2^ C | 284.5 | 0.9 | 25568.38 | HC700 | *sp*^2^ C | 284.5 | 1.05 | 13457.32 |
|  | C-O/C-N | 286.35 | 1.11 | 596.5 |  | C-O/C-N | 285.94 | 1.11 | 1227.28 |
|  | π-π* | 290.8 | 2.9 | 1727.31 |  | π-π* | 290.65 | 2.5 | 573.47 |
|  | *sp*^3^ C | - | - | - |  | *sp*^3^ C | 285 | 1.5 | 6991.8 |
|  | O-C=O/CO_3_ | 288.85 | 1.51 | 415.67 |  | O-C=O/CO_3_ | 289.1 | 1.51 | 693.19 |
|  | C=O | 287.39 | 1.51 | 619.4 |  | C=O | 287.09 | 1.51 | 1097.32 |
| HC1000-400-2h | *sp*^2^ C | 284.5 | 0.98 | 22501.48 | HC700-400-2h | *sp*^2^ C | 284.5 | 1.01 | 14764.89 |
|  | C-O/C-N | 286.1 | 1.11 | 638.46 |  | C-O/C-N | 286.17 | 1.11 | 1638.91 |
|  | π-π* | 290.96 | 2.9 | 907.52 |  | π-π* | 290.67 | 2.75 | 947.2 |
|  | *sp*^3^ C | - | - | - |  | *sp*^3^ C | 285 | 1.11 | 6363.06 |
|  | O-C=O/CO_3_ | 289.05 | 1.51 | 542.96 |  | O-C=O/CO_3_ | 288.79 | 1.51 | 769.35 |
|  | C=O | 287.28 | 1.51 | 643.09 |  | C=O | 287.3 | 1.51 | 1224.94 |
| HC1000-400-5h | *sp*^2^ C | 284.5 | 1.17 | 13571.29 | HC700-400-5h | *sp*^2^ C | 284.5 | 1.07 | 14888.53 |
|  | C-O/C-N | 286.26 | 1.11 | 506.92 |  | C-O/C-N | 286.17 | 1.11 | 1518.03 |
|  | π-π* | 290.72 | 2.5 | 177.97 |  | π-π* | 290.69 | 2.5 | 477.82 |
|  | *sp*^3^ C | 285 | 1.11 | 2394.30 |  | *sp*^3^ C | 285 | 1.29 | 8912.09 |
|  | O-C=O/CO_3_ | 288.77 | 1.51 | 570.91 |  | O-C=O/CO_3_ | 288.94 | 1.51 | 1095.01 |
|  | C=O | 287.21 | 1.51 | 520.11 |  | C=O | 287.19 | 1.51 | 1369.15 |
| O 1*s* | | | | | | | | | |
| HC1000 | C-O | 533.82 | 1.5 | 270.49 | HC700 | C-O | 533.92 | 1.62 | 1218.32 |
|  | CO_3_ | 530.59 | 1.83 | 433.44 |  | CO_3_ | 530.5 | 1.83 | 310.48 |
|  | H_2_O/O_2_ | 535.74 | 1.83 | 80.42 |  | H_2_O/O_2_ | 535.84 | 1.83 | 81.69 |
|  | R-C=O | 532.36 | 1.83 | 714.34 |  | R-C=O | 532.46 | 1.55 | 1340.77 |
| HC1000-400-2h | C-O | 533.82 | 1.53 | 854.66 | HC700-400-2h | C-O | 533.82 | 1.43 | 974.05 |
|  | CO_3_ | 530.6 | 1.83 | 754.52 |  | CO_3_ | 530.6 | 1.72 | 759.13 |
|  | H_2_O/O_2_ | 535.74 | 1.8 | 155.47 |  | H_2_O/O_2_ | 535.74 | 1.83 | 203.81 |
|  | R-C=O | 532.36 | 1.83 | 2172.88 |  | R-C=O | 532.36 | 1.83 | 2871.19 |
| HC1000-400-5h | C-O | 533.82 | 1.45 | 1247.27 | HC700-400-5h | C-O | 533.82 | 1.61 | 2255.82 |
|  | CO_3_ | 530.6 | 1.88 | 904.39 |  | CO_3_ | 530.6 | 1.71 | 884.25 |
|  | H_2_O/O_2_ | 535.76 | 1.70 | 187.78 |  | H_2_O/O_2_ | 535.74 | 1.83 | 235.04 |
|  | R-C=O | 532.36 | 1.83 | 3435.67 |  | R-C=O | 532.36 | 1.83 | 4155.01 |
| N 1*s* | | | | | | | | | |
| HC1000 | Pyridine N | 398.84 | 1.72 | 202.38 | HC700 | Pyridine N | 389.25 | 1.55 | 661.05 |
|  | Pyrrolic N | 400.95 | 1.72 | 557.58 |  | Pyrrolic N | 400.72 | 1.55 | 1014.25 |
|  | Graphitic N | 402.91 | 1.72 | 124.12 |  | Graphitic N | 402.72 | 1.72 | 185.97 |
|  | Oxidised N | 404.66 | 1.72 | 28.56 |  | Oxidised N | 404.99 | 1.72 | 111.52 |
| HC1000-400-2h | Pyridine N | 398.44 | 1.72 | 107.54 | HC700-400-2h | Pyridine N | 398.46 | 1.52 | 785.18 |
|  | Pyrrolic N | 400.98 | 1.72 | 483.82 |  | Pyrrolic N | 400.81 | 1.72 | 1301.45 |
|  | Graphitic N | 402.93 | 1.72 | 95.31 |  | Graphitic N | 402.85 | 1.72 | 177.33 |
|  | Oxidised N | 404.66 | 1.72 | 33.25 |  | Oxidised N | 405.06 | 1.72 | 103.25 |
| HC1000-400-5h | Pyridine N | 398.84 | 1.72 | 472.41 | HC700-400-5h | Pyridine N | 398.64 | 1.68 | 775.65 |
|  | Pyrrolic N | 400.70 | 1.72 | 647.91 |  | Pyrrolic N | 400.89 | 1.72 | 1198.41 |
|  | Graphitic N | 402.53 | 1.72 | 113.17 |  | Graphitic N | 402.74 | 1.72 | 163.90 |
|  | Oxidised N | 404.99 | 1.72 | 42.20 |  | Oxidised N | 404.82 | 1.72 | 68.66 |

*The position of the *sp*^3^ component was constrained to +0.5 eV relative to the *sp*^2^ component during the fittings.

** All the components were fitted using a 70:30 Gaussian:Lorentzian line shape GL(30) apart from the *sp*^2^ C 1*s* component where an asymmetric line shape LA (1.2,2.5,5) was used.

**Table S3. Selected electrochemical data of pristine and ball-milled samples**. Slope and plateau capacity values obtained from the first and second charge processes of the pristine and ball-milled HC700 and HC1000 samples cycled at 5 mA g^-1^. Capacity values were determined using the dQ/dV vs. V curves shown in Figure S10. The plateau processes were defined to start when dQ/dV = 400 mAh g^–1^ V^–1^, following a similar approach to that reported in the literature.^1^ The columbic efficiency of the first and second cycles is also shown.

| Sample | Charge Cycle | Capacity/mAh g^-1^  Slope Plateau Total | | | Onset Plateau  Voltage/mV | CE/% |
| --- | --- | --- | --- | --- | --- | --- |
| HC700 | 1^st^ | 114 | 23 | 137 | 105.9 | 53.6 |
|  | 2^nd^ | 108 | 25 | 133 | 106.3 | 89.2 |
| HC700-400-2h | 1^st^ | 151 | 27 | 178 | 114.2 | 52.3 |
|  | 2^nd^ | 146 | 27 | 173 | 114.0 | 87.9 |
| HC700-400-5h | 1^st^ | 227 | 24 | 251 | 109.1 | 50.5 |
|  | 2^nd^ | 218 | 24 | 242 | 112.3 | 88.9 |
| HC1000 | 1^st^ | 142 | 87 | 229 | 94.2 | 67.2 |
|  | 2^nd^ | 139 | 87 | 226 | 93.3 | 93.4 |
| HC1000-400-2h | 1^st^ | 135 | 80 | 215 | 94.5 | 65.1 |
|  | 2^nd^ | 130 | 83 | 213 | 95.1 | 93.2 |
| HC1000-400-5h | 1^st^ | 246 | 8 | 254 | 70.2 | 51.7 |
|  | 2^nd^ | 232 | 8 | 240 | 72.1 | 88.5 |

**Table S4.** **Specific capacity retention values of pristine and ball-milled samples after 100 cycles.** Capacity retention values for long-term cycling of pristine and ball-milled HC700 and HC1000 samples obtained from data in Figure 4(c and d).

| Sample | Cap. retention (in %) after 100 cycles at 50 mA g^-1^ |
| --- | --- |
| HC700 | 94 |
| HC700-400-2h | 100 |
| HC700-400-5h | 62 |
| HC1000 | 95 |
| HC1000-400-2h | 91 |
| HC1000-400-5h | 8 |

**Table S5. EPR simulation results obtained at 10 K.** *g* value, peak-to-peak linewidth ∆H_pp_ and lineshape information obtained from the EPR spectra measured at 10 K shown in Figure 5.

| Pristine samples @ 10K | *g* | ∆H_pp_/G | Lineshape |  |  |  |
| --- | --- | --- | --- | --- | --- | --- |
| HC700 | 2.0024 | 9.5 | L* |  |  |  |
| HC1000 | 2.0044 | 150 | D (1.07) ** |  |  |  |
| Ball milled samples @ 10K | Broad signal | | | Narrow signal | | |
|  | *g* | ∆H_pp_/G | Lineshape | *g* | ∆H_pp_/G | Lineshape |
| HC700-400-2h | 2.0026 | 26.5 | L* | 2.0036 | 4.9 | L* |
| HC700-400-5h | 2.0025 | 72.9 | L* | 2.0026 | 9.12 | L* |
| HC1000-400-2h | 2.0025 | 140 | D (1.03) ** | 2.003 | 6.1 | L* |
| HC1000-400-5h | 2.0027 | 24.9 | D (1.04) ** | 2.0034 | 6.9 | L* |

* L =Lorentzian lineshape

** D= Dysonian lineshape. The value in brackets indicates the asymmetry parameter A/B, where A and B are the amplitudes of the positive and negative parts of the signal.

**Table S6.** **EPR simulation results obtained at RT.** *g* value, peak-to-peak linewidth ∆H_pp_ and lineshape information obtained from the EPR spectra measured at room temperature shown in Figure S13.

| Pristine samples @ RT | *g* | ∆H_pp_/G | Lineshape |  |  |  |
| --- | --- | --- | --- | --- | --- | --- |
| HC700 | 2.0025 | 32 | L* |  |  |  |
| HC1000 | 2.0033 | 210 | D (1.12) ** |  |  |  |
| Ball milled samples @ RT | Broad signal | | | Narrow signal | | |
|  | *g* | ∆H_pp_/G | Lineshape | *g* | ∆H_pp_/G | Lineshape |
| HC700-400-2h | 2.0021 | 54 | L* | 2.0023 | 5.8 | L* |
| HC700-400-5h | 2.0024 | 118 | L* | 2.0031 | 10.3 | L* |
| HC1000-400-2h | 2.0029 | 200 | D (1.01) ** | 2.0026 | 6.5 | L* |
| HC1000-400-5h | 2.0029 | 27.7 | D (1.1) ** | 2.0029 | 6.4 | L* |

* L =Lorentzian lineshape

** D= Dysonian lineshape. The value in brackets indicates the asymmetry parameter A/B, where A and B are the amplitudes of the positive and negative parts of the signal.

**Table S7. EPR simulation of the ex situ results obtained at 10 K.** *g* value, peak-to-peak linewidth ∆H_pp_ and lineshape information obtained from the ex situ EPR spectra measured at 10K.

| Sample@ 10K | Broad signal | | | Narrow signal | | |
| --- | --- | --- | --- | --- | --- | --- |
|  | *g* | ∆H_pp_/G | Lineshape | *g* | ∆H_pp_/G | Lineshape |
| HC700-400-2h @ 0.02 V | - | - |  | 2.0032 | 6.3 | L* |
| HC700-400-5h @ 0.02 V | - | - |  | 2.0029 | 9.6 | L* |
| HC1000-400-2h @ 0.02 V | 2.0041 | 12.5 | L* | 2.0035 | 5.2 | L* |
| HC1000-400-5h @ 0.02 V | - | - |  | 2.0037 | 7.5 | L* |

* L =Lorentzian lineshape

**List of Figures**


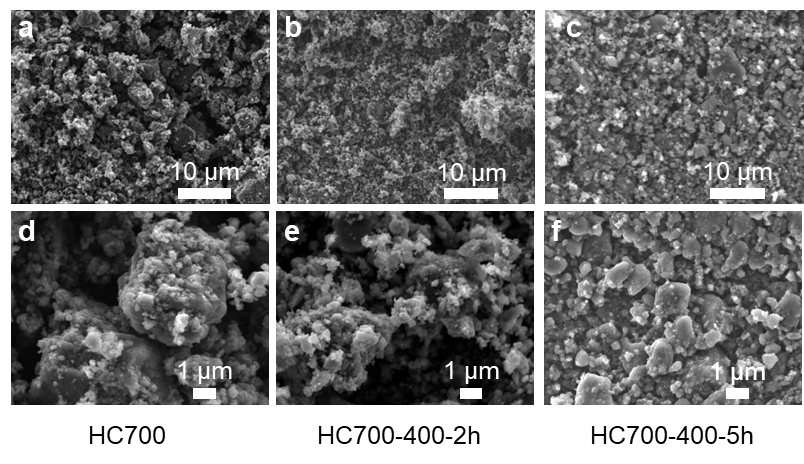


**Figure S1. SEM images of pristine and ball-milled HC700 samples.** SEM images of a,d) HC700, b,e) HC700-400-2h and c,f) HC700-400-5h.


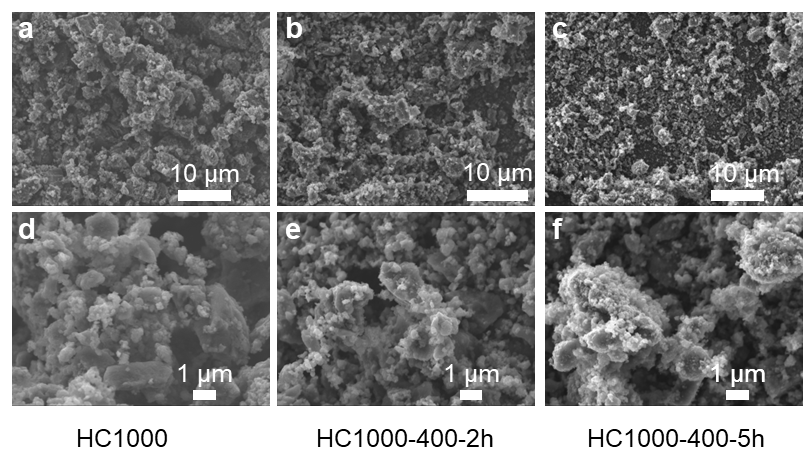


**Figure S2. SEM images of pristine and ball-milled HC1000 samples.** SEM images of a,d) HC1000, b,e) HC1000-400-2h and c,f) HC1000-400-5h.

**
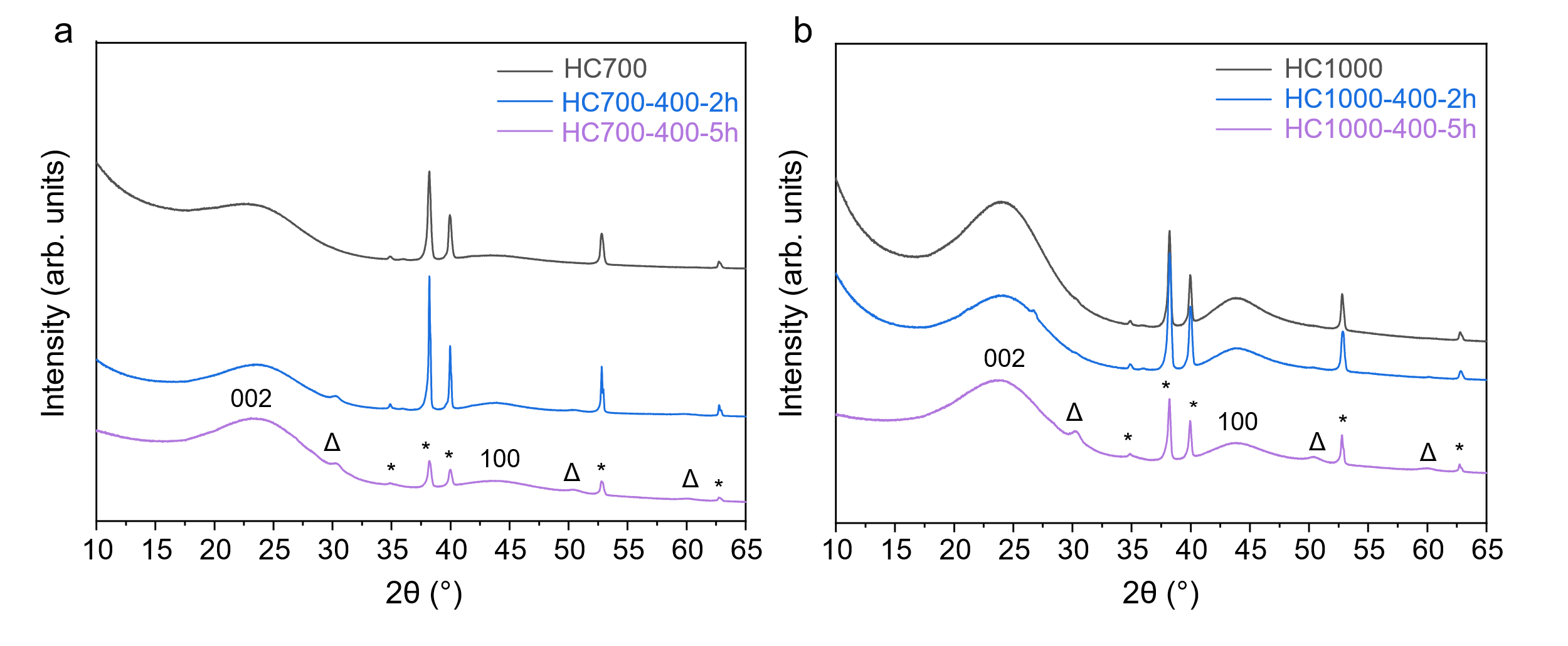
**

**Figure S3. Powder XRD data of pristine and ball-milled HC samples.** (a) HC700 and (b) HC1000 samples. Peaks assigned to the Ti foil have been labelled with a (*) symbol and peaks assigned to zirconium oxide (ZrO_2_) are labelled with a (△) symbol. The ZrO_2_ present in the ball-milled samples is due to the use of ZrO_2_ balls in the ball-milling procedure. Ti reflections are present as Ti foil was used as reference material to obtain accurate 2θ values for the different samples.


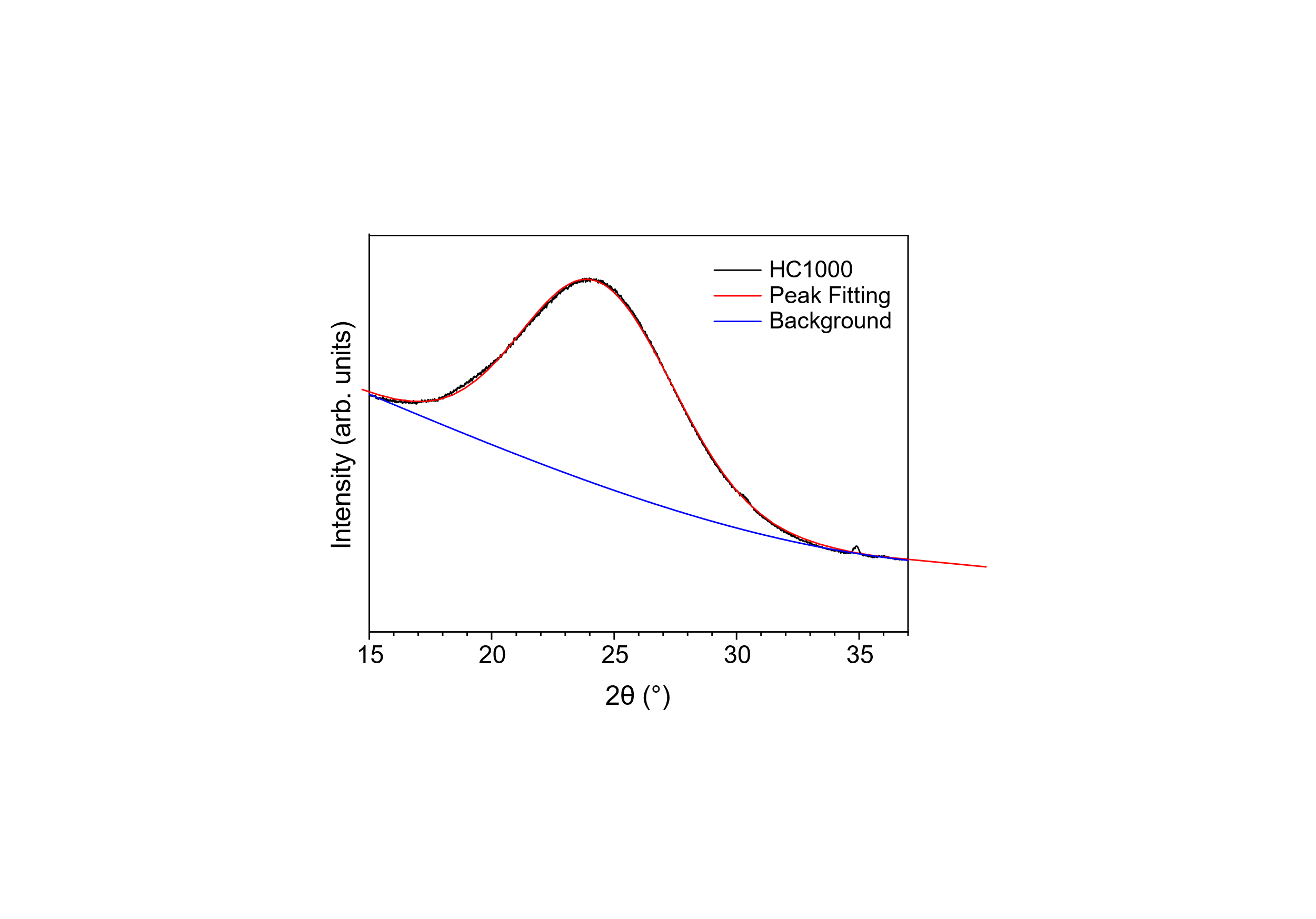


**Figure S4.** **Typical** **fit of the (002) diffraction line.** A fitting of the (002) reflection for pristine HC1000 overlayed onto the experimental XRD data. For the fitting, an asymmetric least squares smoothing baseline was used and a Gaussian peak fit was performed using the OriginPro2022 software package. This type of fitting was performed for all the samples to ascertain the position, FWHM and *d*_002_ values.


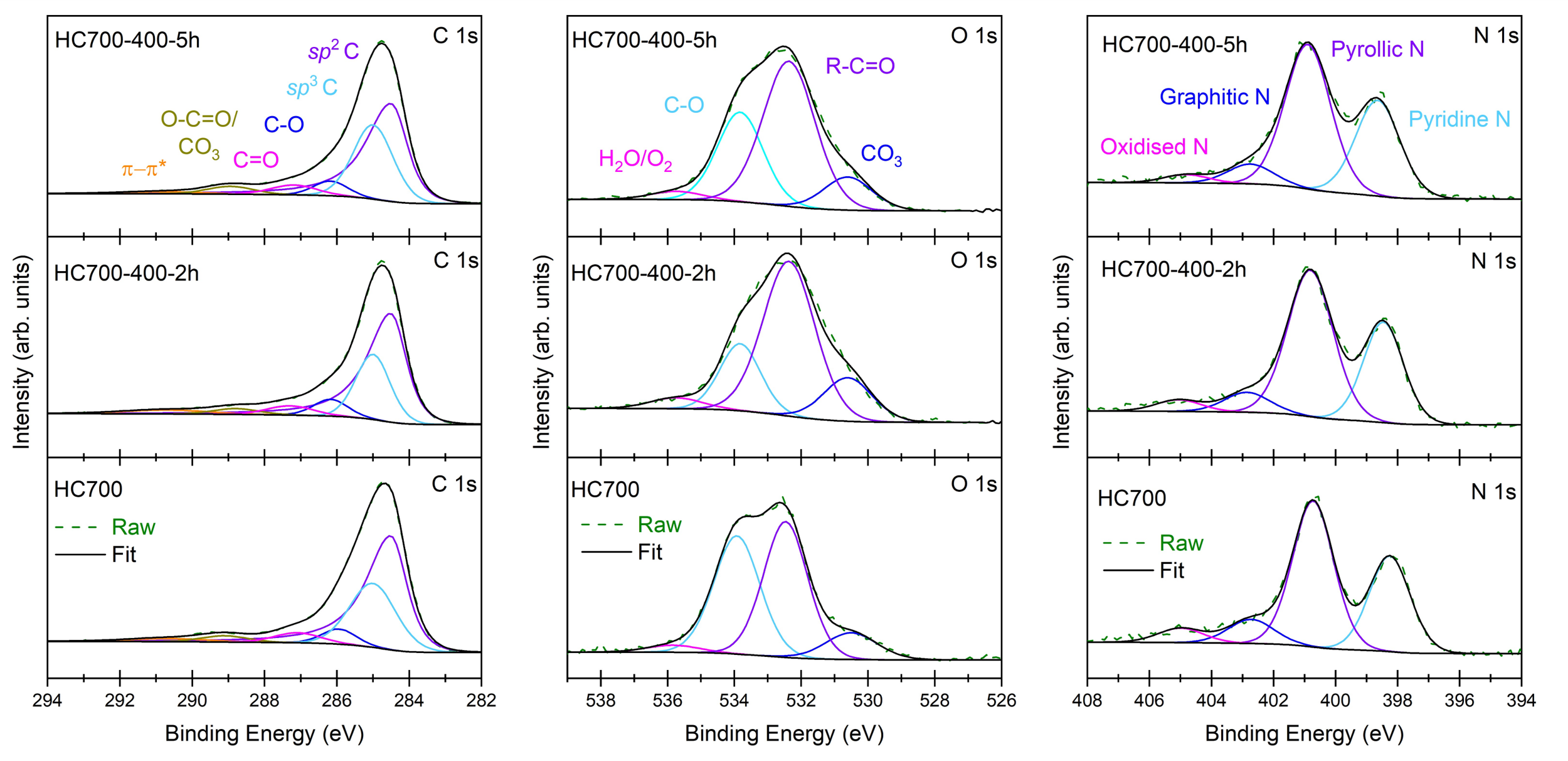


**Figure S5.** **Fitted C 1s, O 1s and N 1s XPS data of pristine and ball-milled HC700 samples.** (a) C 1*s*; (b) O 1*s* and (c) N 1*s* high-resolution XPS data of pristine and ball-milled HC700 samples. High-resolution C 1*s* spectra were deconvoluted into *sp*^2^ C (284.5 eV), *sp*^3^ C (285.0 eV), C-O (286.1 ± 0.2 eV) C=O (287.1 ± 0.2 eV), O–C=O/CO_3_ (288.9 ± 0.2 eV) and π-π* species from *sp*^2^ (aromatic) (290.9 ± 0.2 eV) peaks. ^2, 3, 4, 5^ The O 1*s* spectra were fitted with CO_3_ (530.5 ± 0.1 eV), C=O/R-C=O (532.5 ± 0.1 eV), C–O (533.9 ± 0.1 eV) and ads. H_2_O/O_2_ (535.8 ± 0.1 eV) peaks;^6^ the N 1*s* spectra were fitted with pyridinic-N (398.5 ± 0.3 eV), pyrrolic-N (400.9 ± 0.2 eV), graphitic-N (401.7 ± 0.2 eV) and oxidised-N (404.9 ± 0.2 eV) peaks.^2, 5, 7, 8,^

^
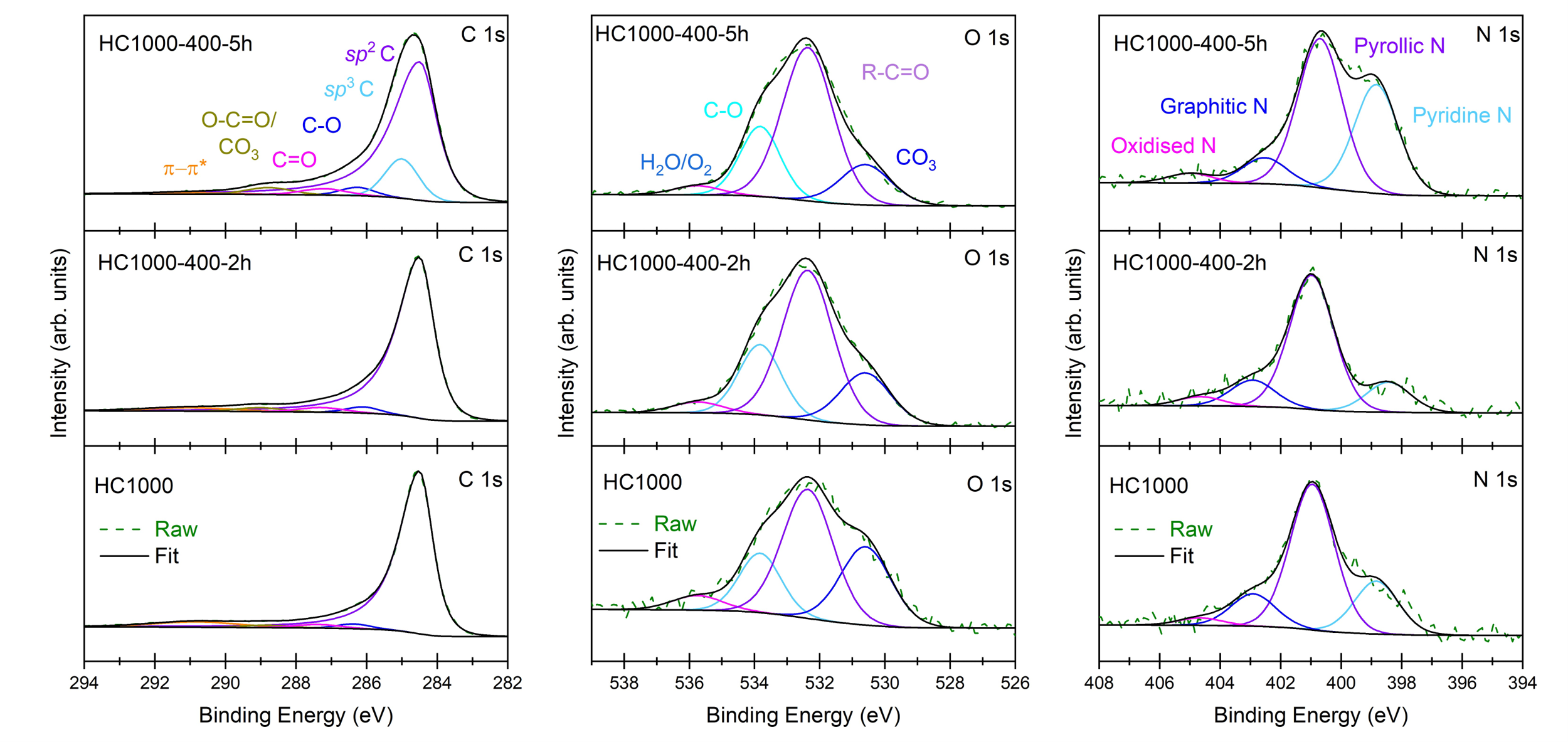
^

**Figure S6. Fitted C 1s, O 1s and N 1s XPS data of pristine and ball-milled HC1000 samples.** (a) C 1*s*; (b) O 1*s* and (c) N 1*s* high-resolution XPS data of pristine and ball-milled HC1000 samples. High-resolution C 1*s* spectra were deconvoluted into *sp*^2^ C (284.5 eV), *sp*^3^ C (285.0 eV), C-O (286.1 ± 0.2 eV) C=O (287.1 ± 0.2 eV), O–C=O/CO_3_ (288.9 ± 0.2 eV) and π-π* species from *sp*^2^ (aromatic) (290.9 ± 0.2 eV) peaks. ^2, 3, 4, 5^ The O 1*s* spectra were fitted with CO_3_ (530.5 ± 0.1 eV), C=O/R-C=O (532.5 ± 0.1 eV), C–O (533.9 ± 0.1 eV) and ads. H_2_O/O_2_ (535.8 ± 0.1 eV) peaks;^6^ the N 1*s* spectra were fitted with pyridinic-N (398.5 ± 0.3 eV), pyrrolic-N (400.9 ± 0.2 eV), graphitic-N (401.7 ± 0.2 eV) and oxidised-N (404.9 ± 0.2 eV) peaks.^2, 5, 7, 8,^


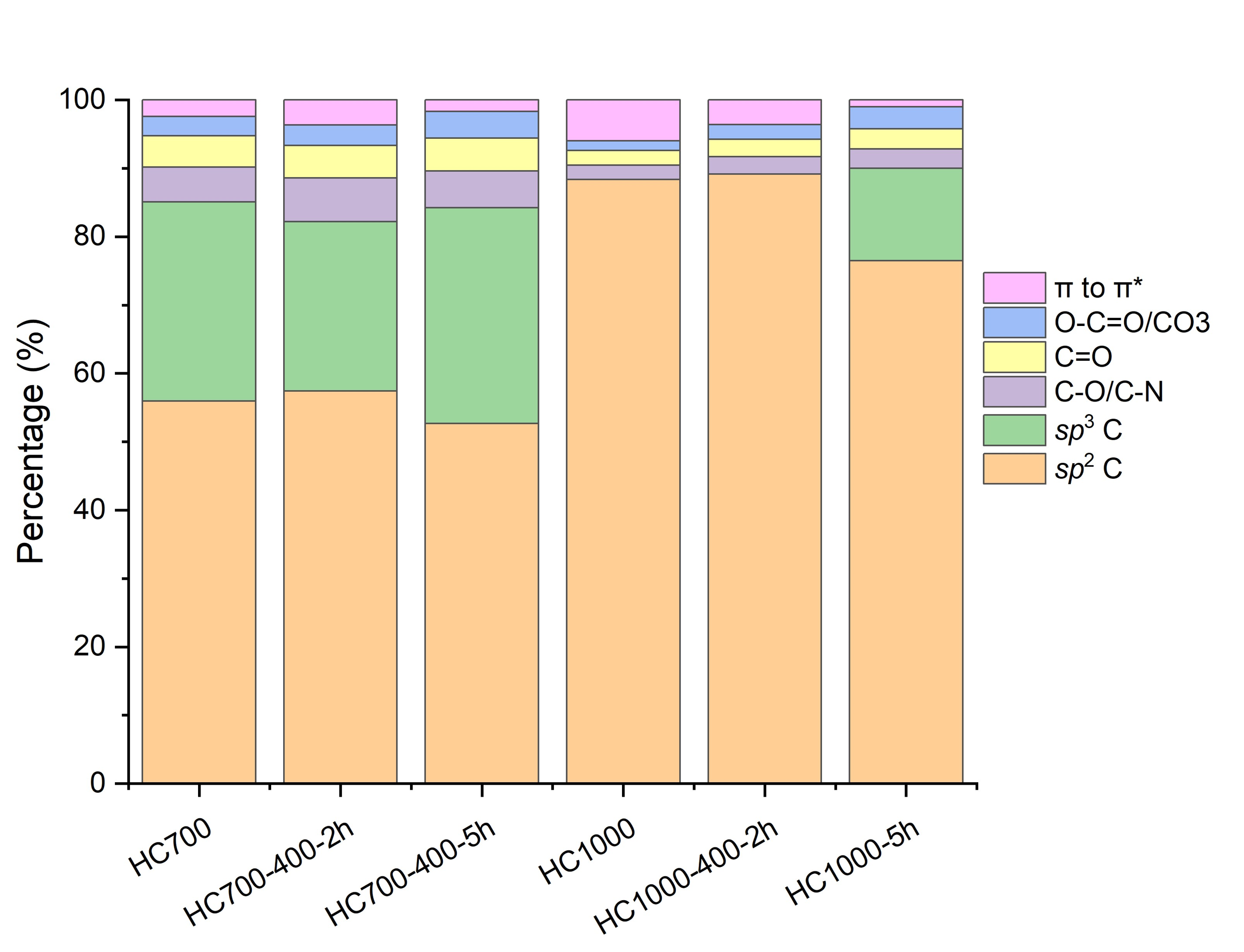


**Figure S7.** **Peak areas obtained from the fittings of the C 1s XPS spectra.** Peak area percentages of individual fitting components shown in the XPS C 1*s* spectra shown in Figures S5(a) and S6(a).


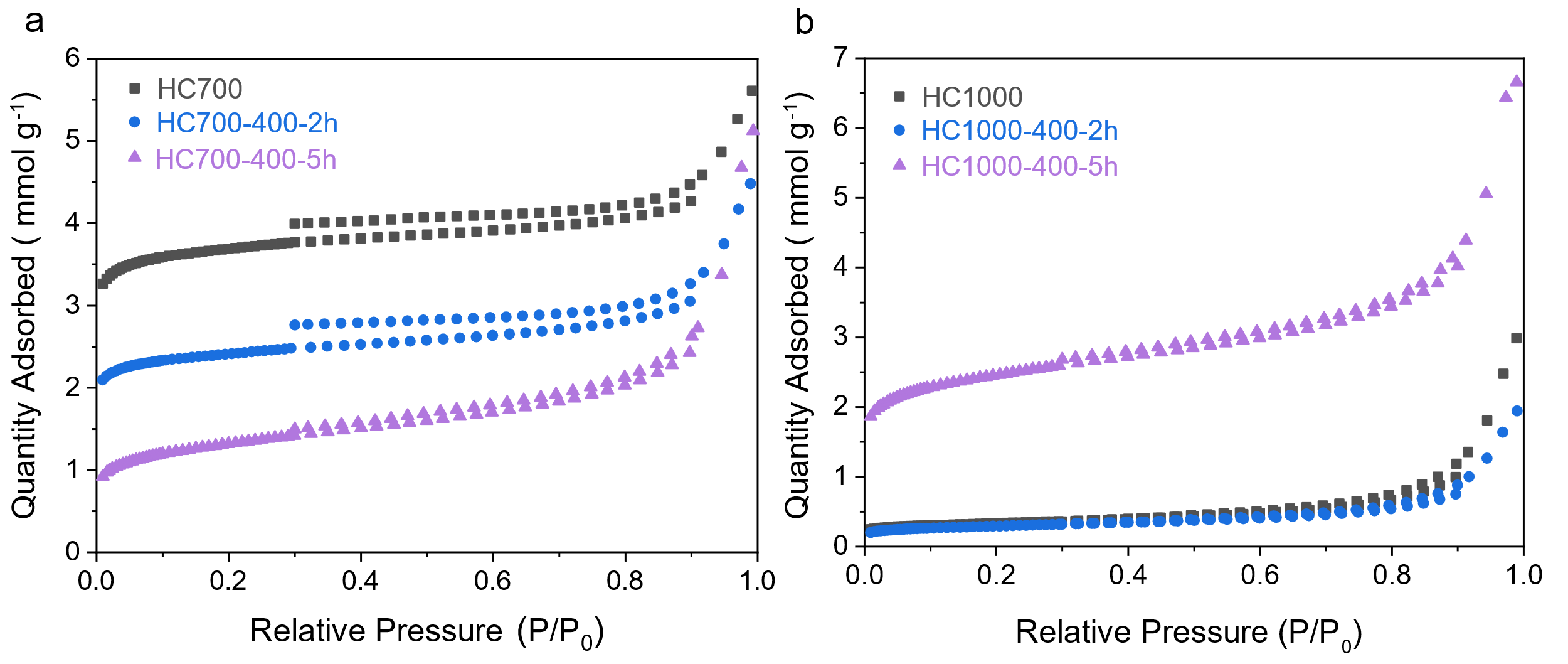


**Figure S8.** **N_2_ adsorption-desorption plots of pristine and ball-milled HC samples.** Brunauer-Emmet-Teller (BET) isotherm plots for N_2_ adsorption-desorption of pristine and ball-milled (a) HC700 and (b) HC1000 samples. Both HC700 and HC1000 ball-milled samples showed Type II isotherms. Specific surface area values are summarised in Table 1.


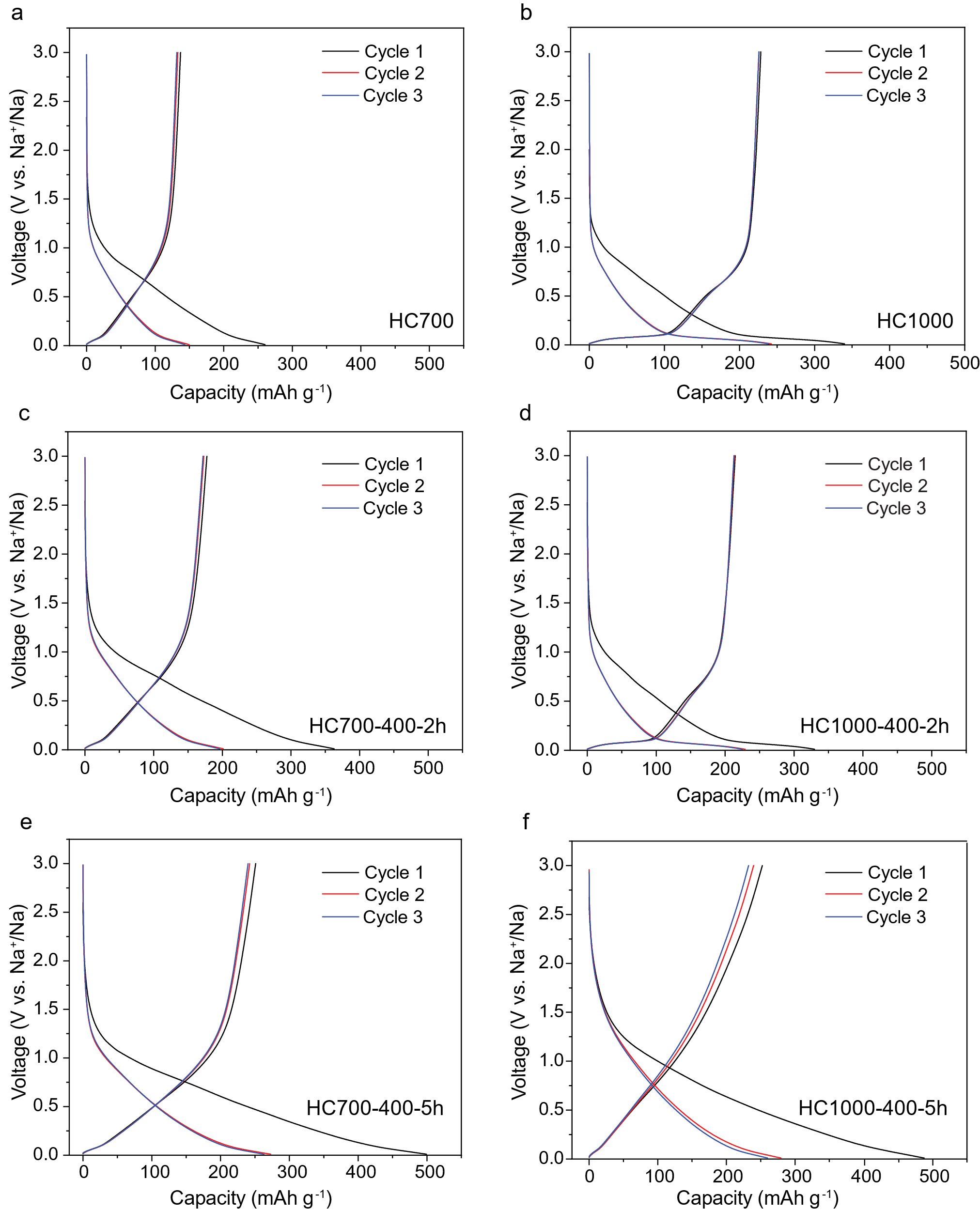


**Figure S9. Load curves of pristine and ball-milled HC samples during cycles 1-3.** Galvanostatic charge-discharge curves of cycles 1-3 for (a, c, e) HC700 and (b,d f) HC1000 pristine and ball-milled samples in the voltage window 3-0.01 V vs. Na^+^/Na at 5 mA g^-1^.


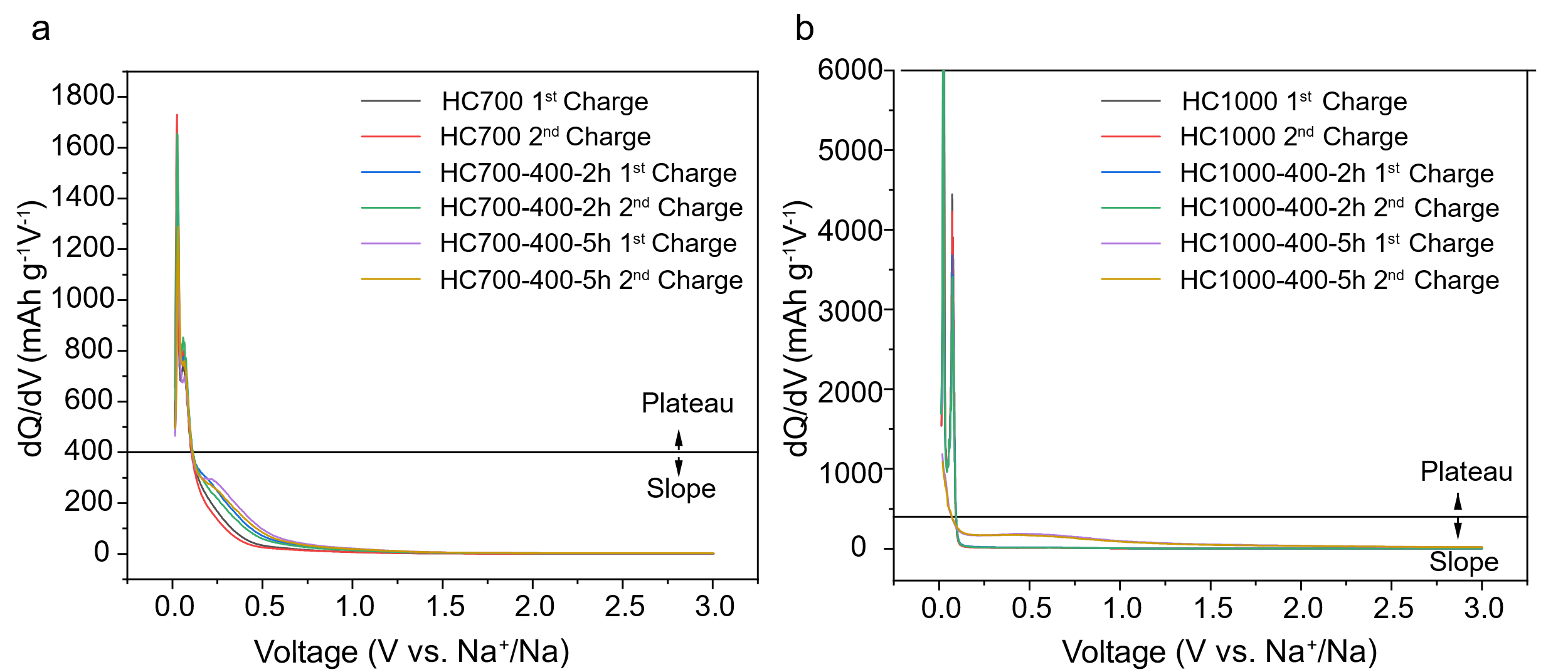


**Figure S10. dQ/dV vs. V plots used to quantify the sloping and plateau capacity contributions of each sample.** dQ/dV vs. V plots of (a) HC700 and (b) HC1000 pristine and ball-milled samples during the first and second charge processes. The corresponding galvanostatic charge-discharge data are shown in Figure S9. The plateau processes were defined to start when dQ/dV = 400 mAh g^–1^ V^–1^ (labelled with a horizontal black line in the plots), following a similar approach to that reported in the literature.^1^


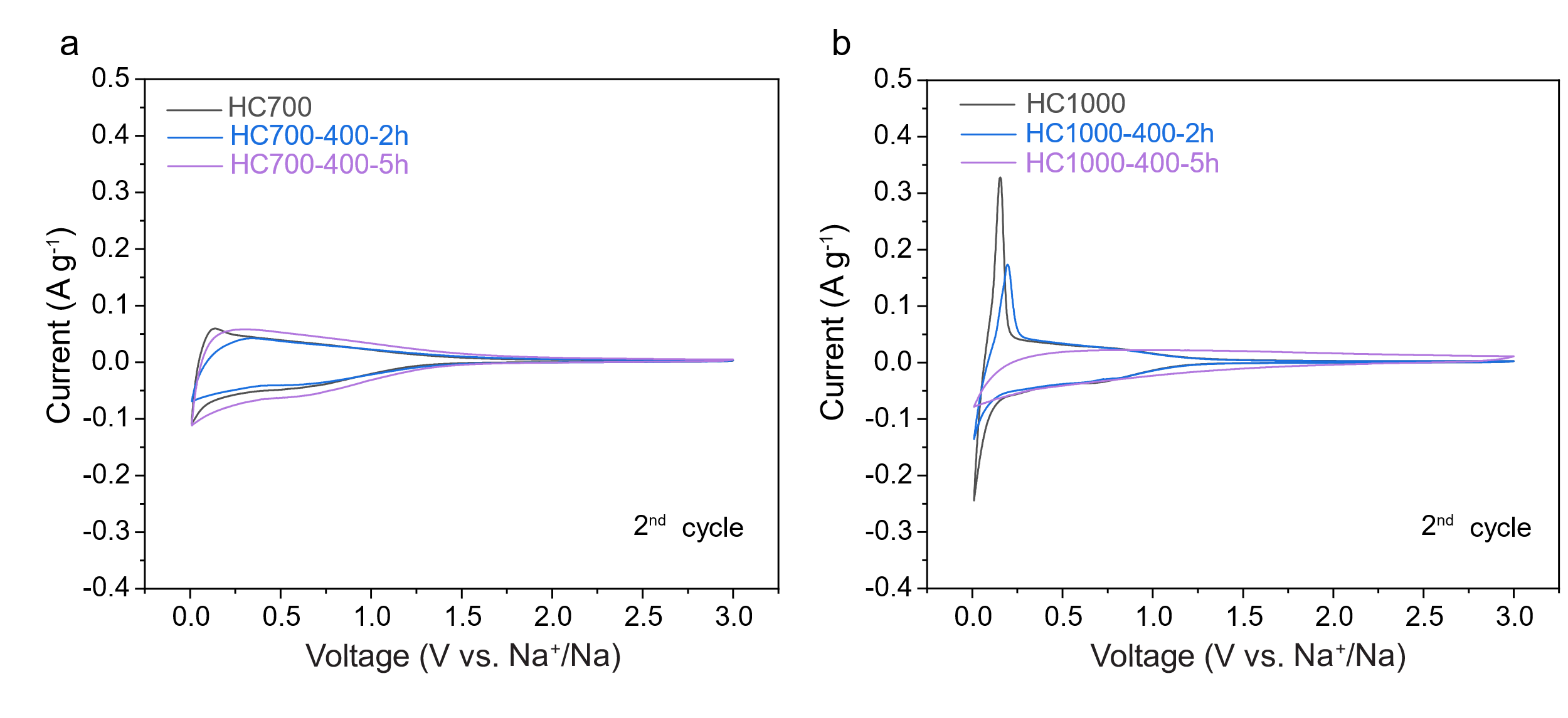
**Figure S11. Cyclic voltammograms showing the second cycle of pristine and ball-milled HC samples**. Second cycle cyclic voltammetry data of pristine and ball-milled (a) HC700 and (b) HC1000 samples in the voltage window 3-0.01 V vs. Na^+^/Na at 0.1 mV s^-1^.


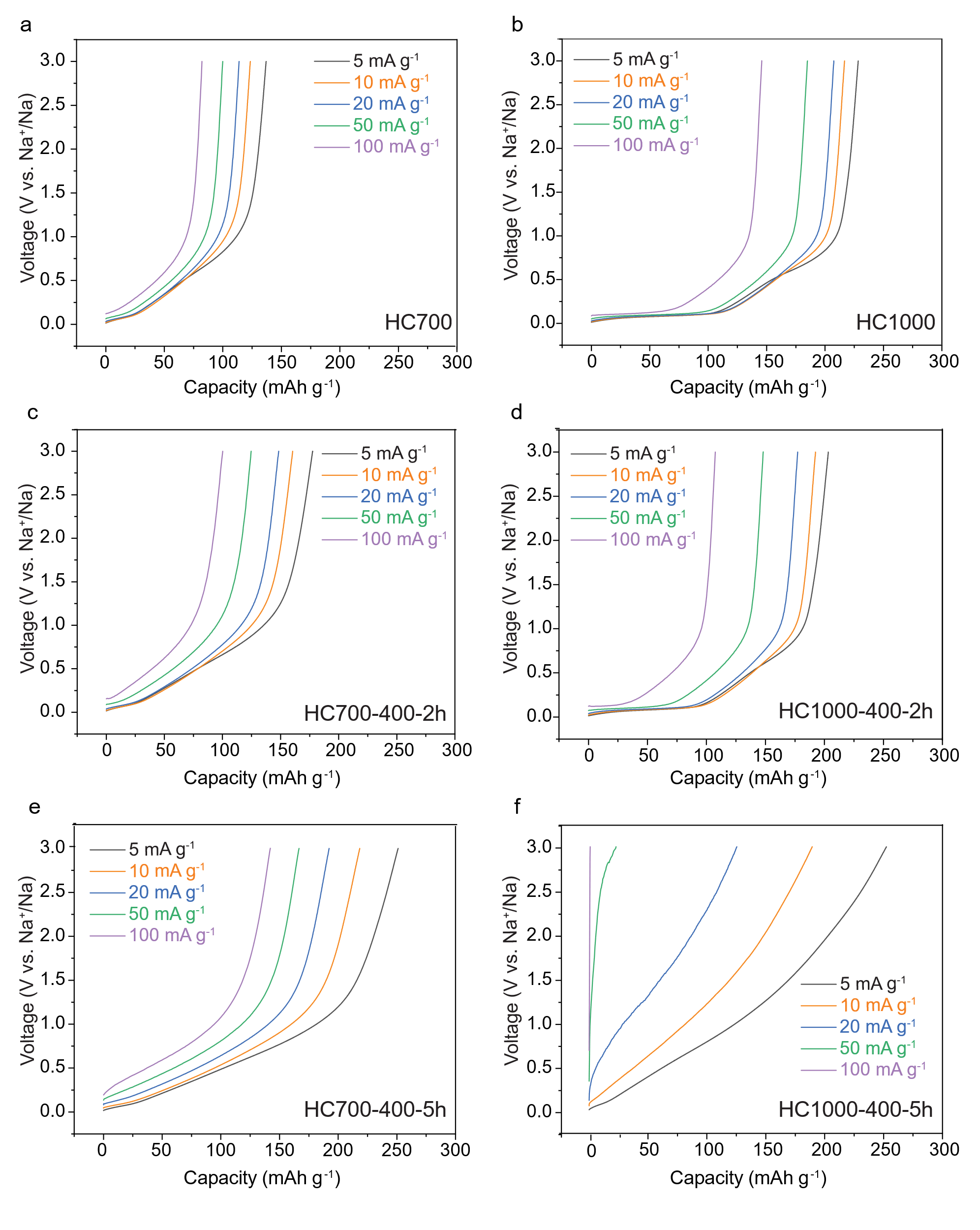


**Figure S12.** **First charge capacity data at 5, 10, 20, 50 and 100 mA g^-1^ current densities.** First charge curves of the HC700 (a, c, e) and HC1000 (b, d, f) pristine and ball-milled samples in the voltage window 3-0.01 V vs. Na^+^/Na at different current densities.


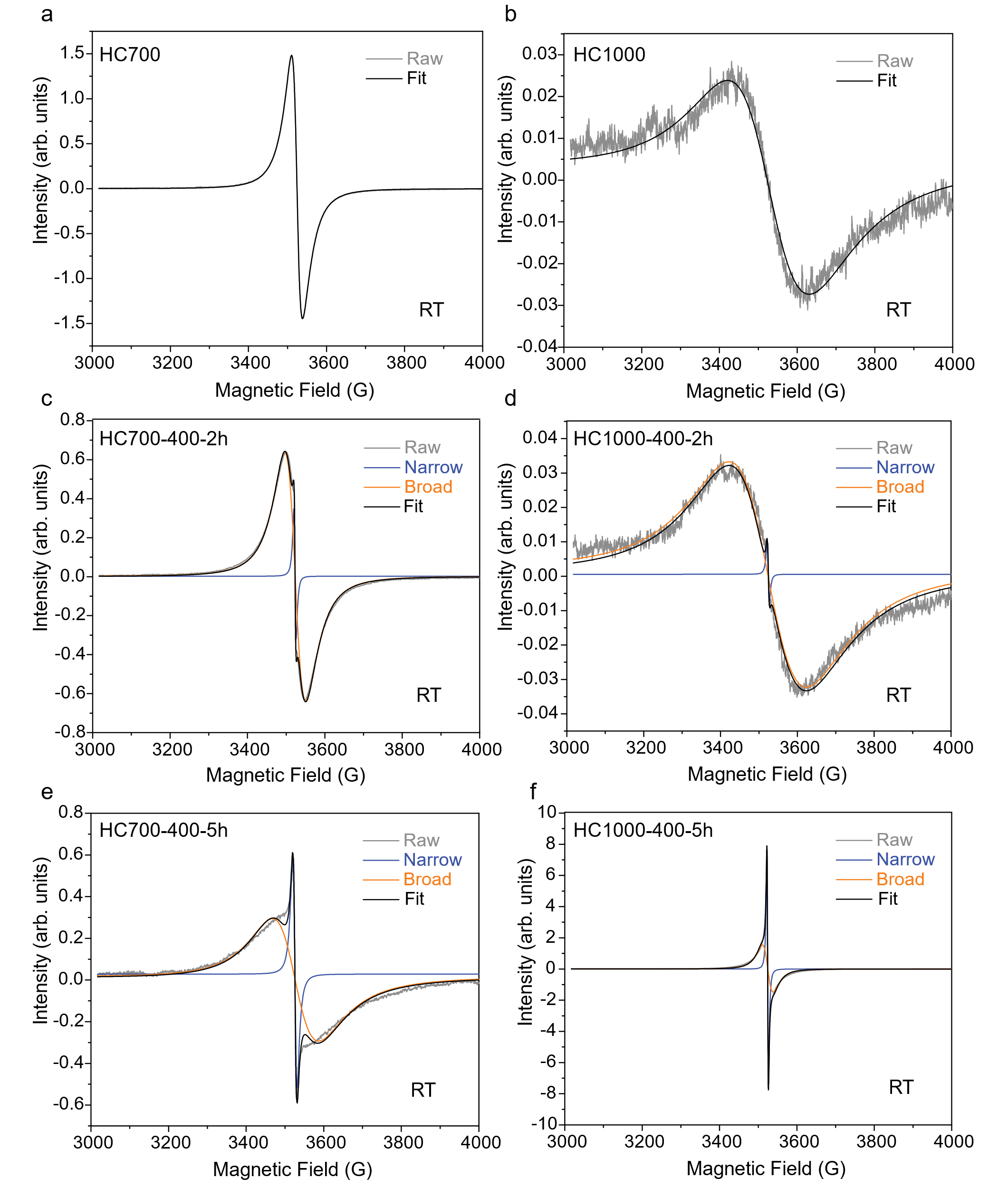


**Figure S13.** **EPR data of pristine and ball-milled HC samples at room temperature.** Fitted EPR spectra at room temperature showing HC700 (a,c,e) and HC1000 (b,d,f) pristine and ball-milled samples using a mass loading of 1 mg. The broad signal (orange) is related to the initial extended aromatic structure in the ball-milled samples, while the narrow signal (blue) appeared after ball-milling.


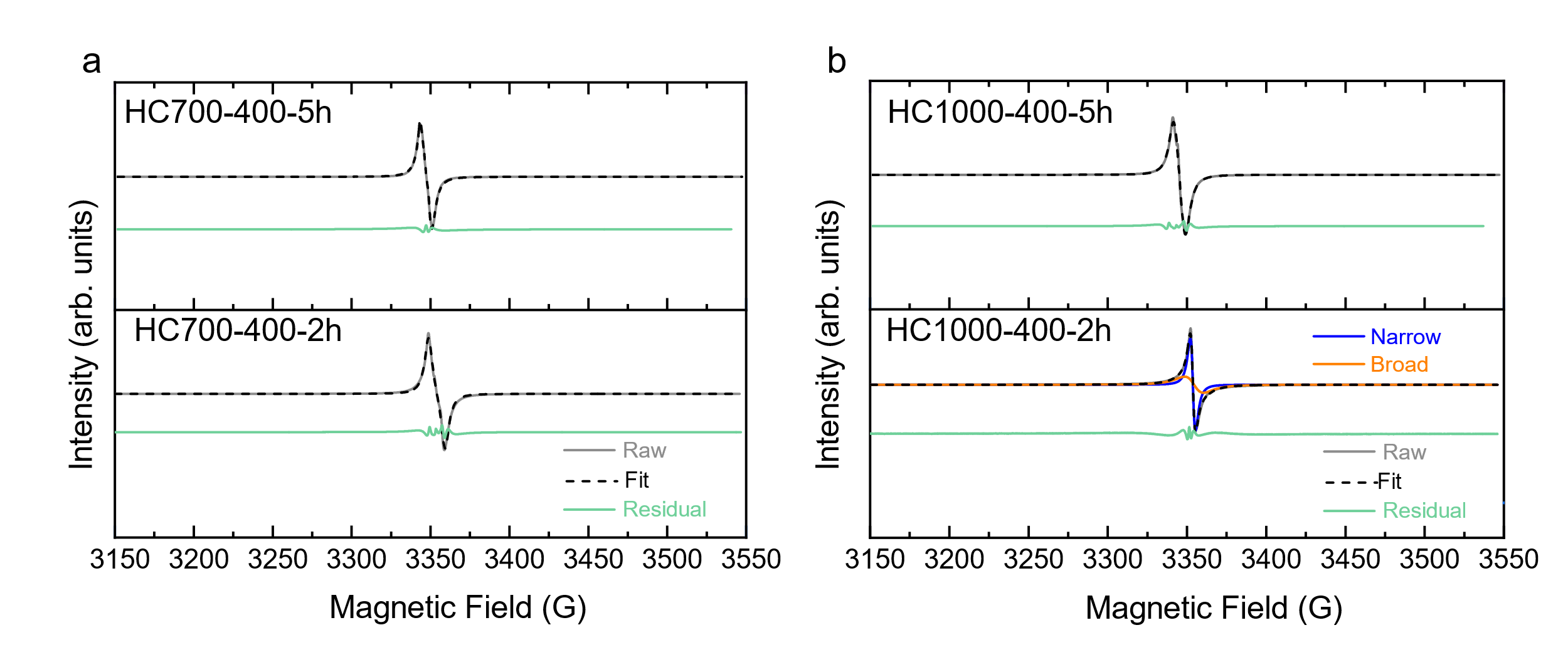


**Figure S14. Ex situ EPR data of ball-milled HC electrodes discharged to 0.02 V.** Fitted EPR data of (a) HC700 and (b) HC1000 electrodes discharged to 0.02 V.

**Supplementary References**

1. Stratford, J.M.*, et al.* Correlating local structure and sodium storage in hard carbon anodes: insights from pair distribution function analysis and solid-state NMR. *J. Am. Chem. Soc.* **143**, 14274-14286 (2021).

2. Wang, B., Likodimos, V., Fielding, A.J., Dryfe, R.A.W. In situ electron paramagnetic resonance spectroelectrochemical study of graphene-based supercapacitors: Comparison between chemically reduced graphene oxide and nitrogen-doped reduced graphene oxide. *Carbon* **160**, 236-246 (2020).

3. Yang, D.*, et al.* Chemical analysis of graphene oxide films after heat and chemical treatments by X-ray photoelectron and Micro-Raman spectroscopy. *Carbon* **47**, 145-152 (2009).

4. Stankovich, S.*, et al.* Synthesis of graphene-based nanosheets via chemical reduction of exfoliated graphite oxide. *Carbon* **45**, 1558-1565 (2007).

5. Zou, Y., Kinloch, I.A., Dryfe, R.A.W. Nitrogen-doped and crumpled graphene sheets with improved supercapacitance. *J. Mater. Chem. A* **2**, 19495-19499 (2014).

6. Dinh, C.T.*, et al.* Multi-site electrocatalysts for hydrogen evolution in neutral media by destabilization of water molecules. *Nat. Energy* **4**, 107-114 (2019).

7. Guo, H.L., Su, P., Kang, X., Ning, S.K. Synthesis and characterization of nitrogen-doped graphene hydrogels by hydrothermal route with urea as reducing-doping agents. *J. Mater. Chem. A* **1**, 2248-2255 (2013).

8. Zou, X., Lou, S., Yang, C. et al. Catalytic Oxidation of NO on N-doped Carbon Materials at Low Temperature. *Catal. Lett.* **151**, 487–496 (2021).
